# Oral Piercing: A Pretty Risk—A Scoping Review of Local and Systemic Complications of This Current Widespread Fashion

**DOI:** 10.3390/ijerph20095744

**Published:** 2023-05-08

**Authors:** Giuseppina Malcangi, Assunta Patano, Giulia Palmieri, Lilla Riccaldo, Carmela Pezzolla, Antonio Mancini, Alessio Danilo Inchingolo, Daniela Di Venere, Fabio Piras, Francesco Inchingolo, Gianna Dipalma, Angelo Michele Inchingolo

**Affiliations:** Department of Interdisciplinary Medicine, University of Bari Aldo Moro, 70121 Bari, Italy

**Keywords:** piercing, oral piercing, oral cavity, enamel fissure, enamel cracks, lip piercing, tongue piercing, complication, review

## Abstract

Piercing insertion is a common practice among people of all ages for different reasons (e.g., esthetics, culture, religion). In the oral cavity, the jewel can be placed in the lips, cheeks, tongue, and frenulum. The presence of an oral piercing could cause local and systemic complications in the short term. In the long term, irreversible damage may occur to the hard and soft tissues of the oral cavity. Different authors in the past have produced publications focusing on this issue. This study analyzes 10 published articles sourced from online databases according to the PRISMA flowchart. These articles were chosen from the 501 suitable papers initially found. PubMed, Web of Science, and Scopus were used as the online databases for searching for papers that matched the topic, using the keywords “complication” and “oral piercing”. The purpose of this review is to describe and analyze all possible complications related to the presence of a piercing in the mouth. Local and systemic complications are related to the presence of plaque and localized pathogenic micro-organisms that can spread via the bloodstream, although they rarely occur in patients without predisposing conditions. Maintaining proper oral hygiene and frequent check-ups are essential to avoid the onset of complications.

## 1. Introduction

The definition of body art is the set of those practices and procedures that use the body as a means of expression and communication.

The most common expressions of this new artistic movement are tattoos and piercings [1].

Focusing on the latter, body piercing is a form of body modification that involves the perforation of different tissues (e.g., cutaneous, subcutaneous, cartilaginous, and so on) to create an opening in which a jewel is placed [2,3].

This practice dates back to ancient civilizations, who used it to signify social status and as a form of embellishment during religious ceremonies. For instance, in Ancient Egypt, researchers found an image of a pierced animal from 1500 BC, and male mummies had pierced nipples as a sign of royal power [4].

Furthermore, Ancient Romans used to insert a ring on adolescents’ prepuces, for the purpose of reducing sexual arousal [5].

During the last century (1970–1980), tattoos and body piercings were no longer associated with religious beliefs, but were considered a form of rebellion and an indication of behavioral issues: there was a widespread idea that people with tattoos and piercings had problems with drugs and smoking, alcohol abuse, anger problems, and aggressive behavior, in part, because of the Punk movement, whose followers had a lot of them [1,6,7,8].

Nowadays, this stereotype has been dismantled: tattooing and piercing are considered an actual form of art, and several parts of the body can be decorated with jewels, mostly for aesthetic reasons and self-expression [9,10,11].

Focusing on the mouth, different studies have shown that the tongue is the most pierced part of the mouth, followed by the lips; lip piercings are usually located laterally on the lower lip (even though piercings can be executed anywhere next to the vermillion border) or in the center of the cupid’s bow [12].

Tongue piercings can be located in the middle (dorsal–ventral) or on the side (dorsal–lateral); the first localization is safer and most commonly executed, as the wound heals within 4 to 6 weeks, faster than other sites, thanks to the continuous movements [13,14,15].

Other sites in which jewels can be placed are the cheeks, frenulum, and uvula [9,16].

**Figure 1:**
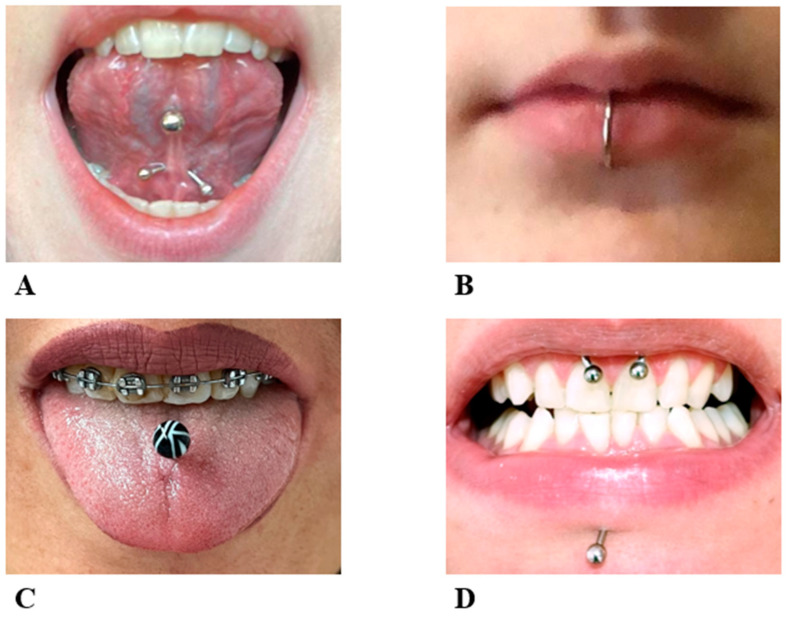
Four patients with different types of oral piercing. (**A**) Lingual frenulum piercing; (**B**) Lower tip piercing; (**C**) Body of the tongue piercing; (**D**) Upper labial frenulum and labret piercing.

Escudero-Castaño et at. distinguish four different types of piercing in the oral cavity: “the barbell”; “the labret”, which is made up of a metallic bar, straight or curved, with a sphere or other geometric figures or jewels at the extremity (Figure 1A–C) [17]; an open-ring ending with a single extremity or double extremity sphere (Figure 1D); and a type in which a powerful magnetic force holds the two studs together [17,18].

Piercing insertion should be considered a proper surgical procedure (even if local anaesthesia is not necessary) and it should be executed under the correct hygienic conditions by properly trained personnel [19,20].

Although piercers use non-toxic and hypoallergenic metals, such as titanium, gold, silver, and stainless steel, they often are not aware of sterilization procedures and the anatomy of the mouth, thus incurring possible complications for the patient’s health [11,20,21].

In general, piercers and the owners of piercings often do not know the proper procedures, the possible intra- and post-operatory complications (both local and systemic), the proper follow-up care, and the procedure for cleaning at home [22,23,24].

Scully and Chen, in 1992, were the first researchers who analyzed the possible problems related to oral piercing [25,26].

In the meantime, many changes have taken place and the art of piercing has spread exponentially; thus, many scientists have written about this matter [4,27,28].

The aim of this review is to analyze the complications reported in the literature occurring in patients with piercings in the oral cavity, in order to present a comprehensive picture of the scientific facts, which could help to increase public awareness of this problem, and to develop and implement effective prevention measures. This is a serious concern for dentists and healthcare workers, who often have patients unaware that their problems may be caused by the presence of their piercing; thus, recognizing the possible complications and risks is important in order to help prevent them [11,29,30].

## 2. Materials and Methods

### 2.1. Protocol and Registration

This current review was carried out in compliance with the standards of the PRISMA Extension for Scoping Reviews (PRISMA-ScR) [31].

### 2.2. Search Processing

A search of the online databases Pubmed, Scopus, and Web of Science was performed for the period from January 2018 to January 2023. The search strategy used the Boolean keywords: “complication” AND “oral piercing” (Table 1). This review used the PICO strategy:

Participants: humans, with no restriction of age and sex

Intervention: presence of piercing in oral cavity

Comparison: absence of piercing

Outcome: complication

### 2.3. Inclusion Criteria

The inclusion criteria:

- Studies only on humans with piercing in the oral cavity;

- English language

- Full text available;

- Clinical trials or case reports.

### 2.4. Exclution Criteria

The exclusion criteria:

- Piercing applied on other parts of the body outside of the mouth;

- In vitro studies;

- Studies performed on animals;

- Review, narrative review and meta-analysis.

### 2.5. Data Processing

During the screening stage, after reading the title and abstract, articles that did not fit the inclusion criteria were excluded. Then, the full text of the remaining articles were read to conduct an eligibility analysis based on the inclusion criteria. Disagreements between the authors on article selection were discussed and resolved.

## 3. Results

A total of 501 articles were found by entering the keywords “oral piercing” and “complication” on three databases, including PubMed (from which 263 articles were found), Scopus (from which 139 articles were found), and Web of Science (from which 99 articles were found). We obtained a total of 501 items, which was reduced to 327 articles after removing duplicates (174).

In the next phase, by reading titles and abstracts, the two reviewers (C.P and L.R.) were able to exclude 261 more articles that were not in accordance with the research criteria or were not open-access.

Thus, 77 articles were found to be suitable in the eligibility phase.

**Figure 2:**
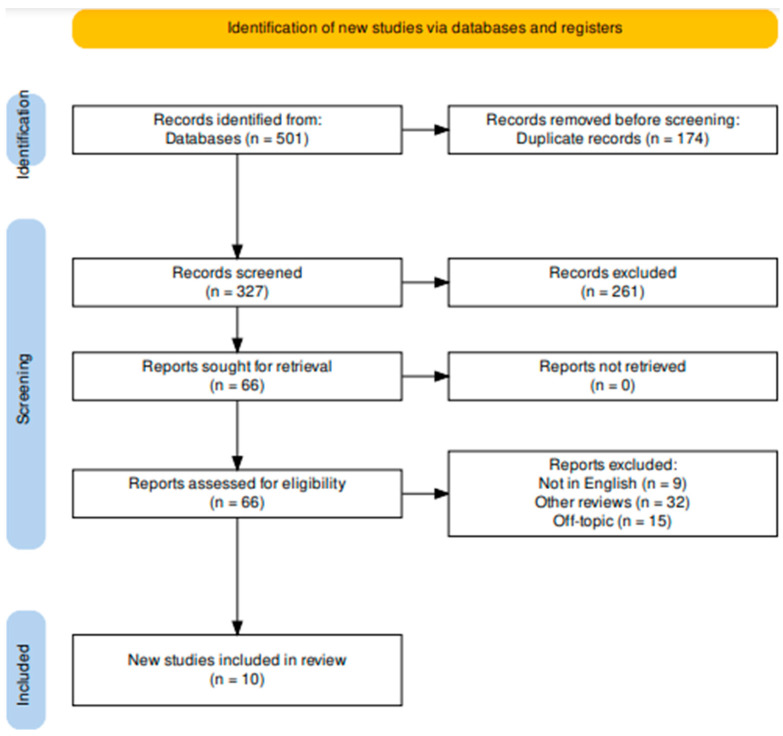
Literature search according to PRISMA Extension for Scoping Reviews (PRISMA-ScR) flow diagram.

The full texts were then read and analyzed. Papers not written in English (nine), other reviews (thirty-two), and articles not related to the topic (fifteen) were excluded. Thus, 10 reports were deemed suitable for inclusion in this review (Figure 2). These were examined and compared (Table 2).

## 4. Discussion

Although oral piercings seem to be a very popular trend nowadays, there are no known accurate statistics on their prevalence in the global population. Several studies have tried to extrapolate statistics regarding the presence of oral piercings in the global population, but their prevalence is highly dependent on geographic localization, traditions, beliefs, social status, and level of instruction [40].

One study conducted on college students revealed that 47 of 454 respondents (10.4%) reported having a tongue piercing [41]. In a German registry of patients with head and neck piercings, 92 of 273 (33.7%) had it on the tongue [42]. In another survey conducted on children, adolescents, and adults with congenital heart disease, 43% of respondents reported having ear piercings but not intraoral piercings [43].

Similarly, the rate of bacterial infections associated with tongue piercings is not well known, but a co-presence of the two variables has been repeatedly found [14].

For this very reason, most dental associations have spoken out against its use, given the possibility of a large number of associated complications, both oral and systemic in nature. Such complications are often unknown and/or underestimated by patients, so the dentist’s role is crucial in informing and preparing patients to recognize and prevent them [30].

### 4.1. Local Complications

Complications affecting the oral cavity can be divided into post-operative complications, which are the direct consequence of the surgical act of piercing insertion, and long-term complications [26,28] (Table 3).

Boardman et al. affirmed that, statistically, the cheeks are more prone to complications compared to the tongue [44].

#### 4.1.1. Local Post-Operative Complications

In a retrospective study involving 108 patients aged 14–39 who had oral piercings for approximately one year, 96% reported post-operative local complications [13,45,46].

The first of these was the beneficial bleeding of the affected area (90%). Important blood vessels run close to the tongue that, if injured, could cause excessive bleeding [47,48,49]. This problem generally arises more for those with coagulation disorders or on anticoagulant or antiplatelet drug therapy [30].

The other complications concerned the presence of perilesional edema for 3 ± 2 days after insertion (80%), the presence of persistent mucosal atrophy (70%), the appearance of erythema on the palatal mucosa (15%), and the appearance of dentinal hypersensitivity (15%). It was also been claimed by patients that they experienced a change in taste perception(dysgeusia) for the next 7–10 days and neurological problems with numbness (related to nerve compression due to edema or, rarely, lesions), particularly on the lingual dorsal lateral side of the tongue, because of the rich presence of motor and sensory fibers [13].

#### 4.1.2. Long-Term Local Complications

**Figure 3:**
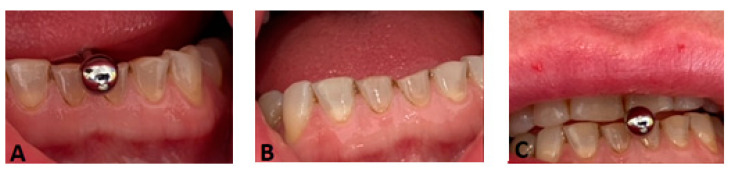
(**A**–**C**): Tooth damage caused by the presence of an oral piercing. Dental damage can be observed on elements 2.1, 4.1, and 3.1. (**A**) Habit of pushing the piercing against the teeth; (**B**) Dental damage on elements 2.1, 3.1 and 4.1 caused by piercing; (**C**) Habit of clamping the piercing between the teeth.

Several of the examined studies found a strong correlation between the presence of an oral piercing and increased incidences of enamel fissures, enamel fractures, and gum recessions (particularly on the lingual side of the incisors) [13,16,28] (Figure 3A–C).

The case-control study conducted by Dirk Ziebolz demonstrated the long-term negative effects of tongue piercings by comparing a group of 46 male subjects (average age 22.1 years) who are members of the German Federal Armed Forces with tongue piercings, and a matched control group of 46 volunteers. The purpose of this study was to highlight the possible damage to dental or periodontal tissue in subjects with tongue piercings. The results showed a statistically significant difference (*p* < 0.001) between the two groups. This demonstrates that tongue piercings are associated with an increase in enamel fissures, enamel cracks, and lingual recessions [13]. Frequently, the habits related to the presence of a piercing (biting, rolling, stroking, sucking, etc.) are the real cause of oral structural defects [50]. In particular, the tongue is the piercing site most strongly associated with the presence of gingival recessions [26,51,52].

Hickey et al. conducted an analysis of the different materials from which piercings are made, and the possible correlation with gingival recessions and the chipping of teeth: it was observed that titanium piercings caused gingival recessions in 52.9% of the evaluated patients, and chipped teeth in 35.7% [33].

Those made of stainless steel caused recessions in 23.5% and chipping in 42.9% of patients.

In contrast, those made of Teflon caused recessions in only 9% of cases and chipping in 14.3%, proving to be the least harmful to the oral cavity [33].

A study conducted at the Department of Oral Sciences and Nano and Biotechnology of the University “G. d’Annunzio” by Chieti has also shown how piercings on the lips or tongue determine a change in salivary composition, in particular, an increase in lysozyme and a more basic pH value [29]. This study considered a sample of 25 adults, 11 men and 14 women, with an average age of 23 years and at least one lip or tongue piercing. Saliva samples were analyzed in the laboratory and they completed a questionnaire before and 72 h after piercing removal. Laboratory tests revealed statistically significant increases in interleukin-8, lysozyme, and amylase (*p =* 0.05). In addition, a more basic pH value (*p* = 0.05) and a decrease in immunoglobulins, particularly in blood clots, were found [53].

No less important is the role of oral hygiene, especially with the presence of a metal object that is an excellent breeding ground for the micro-organisms of bacterial plaque and tartar, which can cause pain, infection, and bad breath.

The *Journal of Adolescent Health* (January 2011) published an article that found that plastic piercings were less colonized by bacteria than ones made from stainless steel [30].

A decline in hygienic conditions is directly proportional to the size of the jewel. In particular, the development of anaerobic bacteria such as A. actinomycetemcomitans, Porphyromonas gingivalis, Prevotella intermedia, Tannerella forsythia and Treponema denticola, which are known to be the main mediators of periodontal pathology [30].

Smoking seems to play a primary role in plaque accumulation [53].

Moreover, the persistence of the jewel in the cavity could generate difficulties during chewing, deglutition, and talking because of the physical encumbrance.

For this same reason, subjects with temporomandibular disorders should not wear piercings, due to pre-existing difficulties in performing movements [54].

### 4.2. Systemic Complications

It is also well known to the scientific community that periodontal disease-related micro-organisms can end up in the circulatory stream and cause inflammatory focus even at sites far from the oral cavity, potentially causing edema and airway impairment [31], the formation of cerebellar abscesses [32], toxic shock syndrome, glomerulonephritis, endocarditis, septic arthritis and Ludwig’s angina [33], cross-transmission of infections such as hepatitis B, C or D and AIDS, the occurrence of metal allergies, and eczematous rash (Table 4).

#### 4.2.1. Cross-Transmission of Infections Such as Hepatitis B, C, or D and AIDS

The risk of cross-infections transmitted by blood is high, especially in cases where the surgery is carried out in non-sterile environments not subject to accurate health control [15,41,42].

A case of an immunosuppressed patient has been reported in the literature: during the insertion of a piercing, the patient was infected with HSV, which evolved into fulminant hepatitis and led to the death of the subject [55].

#### 4.2.2. Allergies to Metals

Multiple studies have shown a statistically significant correlation between piercings and metal allergies. In a study conducted by Larsson-Stymne et al., it was found that 13% of girls with piercings versus 1% without piercings reacted to nickel and/or cobalt [56,57,58].

#### 4.2.3. Endocarditis

The correlation between endocarditis and the presence of a piercing in the oral cavity has been repeatedly examined. A study conducted by the Center Hospitalier Louis Pasteur, France, examined a case of infectious endocarditis due to N. mucosa in a patient with a tongue piercing [35]. Although the Neisseria has a low pathogenicity and is normally present in the upper respiratory tract of humans, the Neisseria’s presence in mucosa has been reported as a causative factor of several cases of endocarditis [35,36,37].

In addition to the Neisseria, other pathogenic micro-organisms such as Gemella species [38], Staphylococcus aureus, Pseudomonas aeruginosa and group A β-hemolytic Streptococcus [30], and *H. aphrophilus* [39] were analyzed. A study on the latter pathogen was conducted by the Department of Internal Medicine at the Mercer University School of Medicine (Georgia), which demonstrated the correlation between an endocarditis case and *H. aphrophilus*, probably caused by a tongue piercing in a patient with congenital heart disease [39].

However, from the different analyzed studies it is clear that the complication of endocarditis is quite rare and is usually associated with a pre-existing heart condition [44,52,59].

In these cases, it would be best to prescribe antibiotic prophylaxis before performing the manoeuvre, in order to avert possible risks [30].

After the insertion of the piercing, the patient should pay attention to oral hygiene and follow these instructions:

- Apply cold substances near the insertion area to reduce edema;

- Follow a liquid and cold food regimen in the 24 h after surgery; on the following days it is possible to eat soft food;

- Avoid spicy or hard foods, chewing gum, alcohol, and caffeine for at least one week;

- Do not rinse the mouth for 24 h post-operation, and then use mouthwash with chlorhexidine 0.12% 3/4 times a day for at least a week;

- Apply a chlorhexidine gel that decreases inflammation;

- Avoid onychophagy, titillophagy, and other vicious habits that can cause micro-trauma to the mucosa;

- Clean the jewel daily with a soft toothbrush, removing all plaque residue;

- Replace the piercing with a smaller one once the wound is healed.

Periodic checks performed by dentists and dental hygienists play a fundamental role in the early diagnosis of possible complications; however, the main role is played by the patient and their compliance in keeping the piercing clean [19,30].

The limitations of our scoping review are related to the randomization that may have been conducted incorrectly, the variable quality of the studies, and the fact our analysis did not take into account that piercing surgery may be operator-dependent and different surgical protocols may be used depending on the site in the oral cavity in which the jewels are placed.

## 5. Conclusions

Although piercing insertion has been a widely used practice since ancient times, nowadays, the information regarding the possible complications that patients might incur in the short and long term is still poor.

As can be seen from the articles included in this review, even if complications first occur in the insertion phase due to a lack of anatomical knowledge and hygienic norms by the piercer and errors during the insertion phase, the main problem is that patients are not accurately informed about the maintenance and home hygiene care of the jewel.

This results in plaque accumulation and colonization by potentially pathogenic micro-organisms that cause local inflammation.

If the inflammatory trigger is not removed in time and the infection spreads through the bloodstream, the systemic complications incurred could be potentially life-threatening, although the studies reviewed state that this is very rare and mainly affects individuals with predisposing medical conditions.

In addition, the persistence of the piercing in the oral cavity can result in continuous injuries to the gums, with consequent recessions and hard tissue injuries to dental elements, which are often irreversible and can cause permanent damage.

It would be better if patients with systemic diseases avoid piercings because of the increased fragility they are subject to. Young people without health problems, on the other hand, have a lower chance of incurring risks; the important thing is that they maintain good local hygiene.

For this reason, it is important that, even before undergoing this procedure, the patient receives the correct information and instructions regarding hygiene and the maintenance of the piercing, since the problems are often underestimated by the patient themself.

## Figures and Tables

**Table 1:** Database search indicator.

Article screening strategy	Database: Pubmed, Scopus, Web of Science
	Keywords: A “oral piercing” and B “complication”
	Boolean variable: AND
	Timespan: 2018–2023
	Language: English

**Table 2:** Included studies that explored local and systemic complications.

Authors	Type of Study	Object	Study Design and Timeline	Result
C.H. Yu et al. [32]	Case report	Relationship between the presence of oral piercing and endocarditis	Reports a case of prosthetic valve endocarditis caused by a Gemella species in a patient with a pierced tongue.	Illustrates that bacterial infections associated with tongue piercing are an emerging complication.
B.M. Hickey et al. [33]	Epidemiological survey	Identify the types and rate of post-piercing complications.	A questionnaire was submitted to 201 pierced people attending the University of Strasbourg dental hospital.	More than 23% of those wearing a piercing had suffered some form of complication.
A. Plessas et al. [34]	Cross-sectional study	Analyze the prevalence of oral piercing complications in dental and periodontal tissues	110 pierced subjects without systemic disease or condition, subjected to a clinical examination.	About one-third of the dental elements adjacent to the piercings show abnormal tooth wear and/or chipping/cracking
H. Tronel et al. [35]	Case report	Relationship between a case of endocarditis and presence of oral piercing	Report a case of infective endocarditis due to N. mucosa that probably resulted from tongue piercing.	Oral Piercing is generally not regarded as a risk factor for endocarditis.
H. Akhondi et al. [36]	Case report	Relationship between a case of endocarditis and presence of oral piercing	*H. aphrophilus* endocarditis possibly caused by tongue piercing in a patient with congenital heart disease	23% of patients had piercing-related infections but no endocarditis was reported in that study.
D. Tripodi et al. [37]	Cross-sectional study	Evaluate variation in the inflammatory or immunity components of the saliva of patients with oral piercings	The saliva of 25 pierced adults was examined and data were statistically analyzed.	Oral piercing determines an increase in saliva enzymes and a more basic pH value.
D. Ziebolz et al. [14]	Case-control study	Analyze the prevalence of oral piercing complications in dental and periodontal tissues	Dental examination of members of the German Federal Armed Forces who had TP (group TP) and a matched control group (group C) volunteered to take part in the study.	Tongue piercing is correlated with an increased occurrence of lingual recessions enamel fissures, enamel cracks.
F. Inchingolo et al. [19]	Retrospective study	Analyze the prevalence of oral piercing complications in dental and periodontal tissues	108 pierced patients aged between 14 and 39 years underwent clinical examination to reveal the possible presence of late complications.	No patients developed widespread complications.
A. Simões et al. [38]	Cross-sectional study	Analyze the prevalence of oral piercing complications in dental and periodontal tissues	109 piercings seen in 82 individuals, who filled a questionnaire and were submitted to an oral examination.	63.3% of the observed piercings have complication/changes associated.
C. Creguta Albu et al. [39]	Case-control study	Association between a tongue piercing and tic behavior	Intra-oral examination of a pierced female patient aged 22	This study demonstrates dental complication associated with tongue piercing and tic behavior

**Table 3:** Oral complication of piercing.

Dental	Other Complications
Tooth fracture restoration Abrasion Tooth sensitivity Pulpal pathology Gingival recession Tooth mobility Electrogalvanism	Pain Ulceration Penetration into tissues Hyperplasia Integration into tissues Rejection and migration Oedema leading to airway risk Inhalation Sialoadenitis

**Table 4:** Systemic complication of oral piercing.

Infective	Neurological and Vascular	Allergies
Endocarditis Oral abscess Cellulites Ludwig’s angina Toxic shock syndrome Cerebellar abscess Glomerulonepheritis Hepatitis (A; B; C; D) Tuberculosis Tetanus Septicaemia Syphilis	Paraesthesia Ageusia Parageusia Hypogeusia Haemorrhage Hypovolemia	Contact dermatitis Eczema

## Data Availability

Not applicable.
